# A Novel Multiplex Tetra-Primer ARMS-PCR for the Simultaneous Genotyping of Six Single Nucleotide Polymorphisms Associated with Female Cancers

**DOI:** 10.1371/journal.pone.0062126

**Published:** 2013-04-17

**Authors:** Chen Zhang, Ying Liu, Brian Z. Ring, Kai Nie, Mengjie Yang, Miao Wang, Hongwei Shen, Xiyang Wu, Xuejun Ma

**Affiliations:** 1 Key Laboratory for Medical Virology, Ministry of Health-National Institute for Viral Disease Control and Prevention, Chinese Center for Disease Control and Prevention, Beijing, China; 2 Department of Food Science and Engineering, College of Science and Engineering, Jinan University, Guangzhou, China; 3 Institute of Genomic and Personalized Medicine, School of Life Science and Technology, Huazhong University of Science and Technology, Wuhan, China; Northwestern University Feinberg School of Medicine, United States of America

## Abstract

**Background:**

The tetra-primer amplification refractory mutation system PCR (T-ARMS-PCR) is a fast and economical means of assaying SNP's, requiring only PCR amplification and subsequent electrophoresis for the determination of genotypes. To improve the throughput and efficiency of T-ARMS-PCR, we combined T-ARMS-PCR with a chimeric primer-based temperature switch PCR (TSP) strategy, and used capillary electrophoresis (CE) for amplicon separation and identification. We assessed this process in the simultaneous genotyping of four breast cancer–and two cervical cancer risk–related SNPs.

**Methods:**

A total of 24 T-ARMS-PCR primers, each 5′-tagged with a universal sequence and a pair of universal primers, were pooled together to amplify the 12 target alleles of 6 SNPs in 186 control female blood samples. Direct sequencing of all samples was also performed to assess the accuracy of this method.

**Results:**

Of the 186 samples, as many as 11 amplicons can be produced in one single PCR and separated by CE. Genotyping results of the multiplex T-ARMS-PCR were in complete agreement with direct sequencing of all samples.

**Conclusions:**

This novel multiplex T-ARMS-PCR method is the first reported method allowing one to genotype six SNPs in a single reaction with no post-PCR treatment other than electrophoresis. This method is reliable, fast, and easy to perform.

## Introduction

The role of single nucleotide polymorphisms (SNP's) in contributing to the variability between individuals in susceptibility to cancer[1], tumor growth and metastasis rate[2]–[4], as well as in treatment efficacy and adverse drug responses, has been well recognized[5], [6]. Among the many methods that have been developed to genotype SNPs, the tetra-primer amplification refractory mutation system PCR (T-ARMS-PCR) has proved to be rapid, simple and economical [7]–[11]. Through combination of two outer primers and two allele-specific inner primers, genotyping requires only a single PCR followed by electrophoresis separation[8]. Multiplex PCR was incorporated in T-ARMS-PCR, using eight primers in one PCR, and is able to simultaneously detect two mutations [9]. Separately, chimeric-primer-based multiplex PCR, which adds a universal 5′ tag to the sequence specific primers for multiple targets, has been reported to improve the throughput and efficiency of the polymerase chain reaction[12]. With its high efficiency in detecting tens of different PCR products in one reaction, the use of chimeric primer PCR has frequently been reported for use in mRNA quantification [13]–[15] and pathogen detection[16], [17].

Breast cancer and cervical cancer have become the most frequently diagnosed cancers and the leading causes of cancer death among females[18]. Recent studies show that somatic variants in susceptibility regions are associated with the likelihood of occurrence of breast and gynecologic cancers [19]–[23]. SNPs were chosen for this study on the basis of reported associations with these cancers and having a reasonably high prevalence in Asian populations. Four low-penetrance variants for the prediction of risk of breast cancer were selected. SNPs rs4784227 [24] and rs3803662 [25] are located in the transcription factor TOX3; rs1219648 lies within FGFR2, which contributes to cell growth, invasiveness, motility, and angiogenesis [26]; rs889312 [27] is within MAP3K1, which is linked to cellular response to mitogens. Two variants associated with risk of cervical or ovarian cancer were selected. SNP rs750749 [21] is a polymorphisms in CD83, which is involved in immune recognition and antigen presentation; rs749292 in CYP19A1, which plays a key role in estrogen biosynthesis [22], [23].

In this paper, we describe a novel multiplex T-ARMS-PCR allowing for the simultaneous genotyping of 6 SNPs (rs4784227, rs3803662, rs1219648, rs889312, rs750749 and rs749292) associated with breast and gynecologic cancers in a single tube using 24 chimeric primers and a pair of universal primers The use of chimeric primers and a temperature switch PCR (TSP) strategy were combined with T-ARMS-PCR to optimize the amplification parameters and improve the throughput of SNP genotyping. The combination of these different genotyping techniques demonstrates for the first time the ability of tetra-primer ARMS-PCR to reliably and efficiently detect six SNPs in a single reaction. Since more than 10 PCR products with different lengths need to be identified, capillary electrophoresis (CE) is used instead of agarose gel electrophoresis.

## Materials and Methods

A total of 186 blood specimens from healthy Chinese female volunteers who were being monitored for potential hypertension were collected at community health centers in Wuhan, China during 2011 for this study. All aspects of the study were performed in accordance with the national ethics regulations and approved by the Institutional Review Boards of the Centre for Disease Control and Prevention 70 of China, as well as the Ethics Committee of Huazhong University of Science and Technology. Participants received “Written Informed Consent” of the study's purpose and of their right to keep information confidential. Written consent was obtained from all participants or their guardians.

Genomic DNA was extracted from 0.2 ml fresh peripheral blood samples by use of the Wizard^®^ Genomic DNA Purification Kit (Promega) according to manufacturer's instructions. Extracted DNA samples had a final concentration ranging from 55–365 ng/µL.

To overcome the limitations of standard multiplex T ARMS-PCR methods, the proposed method was optimized in terms of primer design, PCR cycling conditions and in the utilization of chimeric primers and TSP strategy, as described in our previous reports in the detection of Influenza viruses and human hand foot and mouth associated pathogens[16], [28]. A total of 24 chimeric primers each consisting of a gene-specific sequence with a universal tag sequence at the 5′ end were used. The gene-specific portions of the primers were designed according to the requirements of T-ARMS-PCR. The specificity of allele-specific primers is conferred by the identity of the terminal 3′ nucleotide with either the wild-type or the mutant allele, specificity is increased by the introduction of a deliberate mismatch at position -1 from the 3′–terminus. A pair of universal primers and six sets of T-ARMS-PCR chimeric primers were used for amplification. Detailed primer sequences and working concentrations for each SNP are listed in Table 1.

**Table 1:** Primers for the multiplex tetra-primer ARMS-PCR for SNPs associated with breast and gynecologic cancers.

SNP	Primer	Primer Sequence	Working concentration(nM)	Length (bp)
rs4784227	Forward inner primer(T):	AGGTGACACTATAGAATAAAAAGTCCCAATTTGTAGTGTTTGaT^a^^,^ b ^,^ c	40	322
	rs4784227-476T-2A			
	Reverse inner primer(C):	GTACGACTCACTATAGGGAATGGGAGTATTTACATCACAATAATgG^a^^,^ b ^,^ c	40	297
	rs4784227-527G-2G			
	Forward outer primer:	AGGTGACACTATAGAATACTGACCCCTTTAGACACGG^a^	6.5	
	rs4784227-268			
	Reverse outer primer:	GTACGACTCACTATAGGGAGGGCTTCAACACAGTCAGTTC^a^	6.5	
	rs4784227-760			
rs1219648	Forward inner primer(G):	AGGTGACACTATAGAATACACGCCTATTTTACTTGACACAaG^a^^,^ b ^,^ c	45	269
	rs1219648-278G-2A			
	Reverse inner primer(A):	GTACGACTCACTATAGGGAAGCCATGGCCATCCTTGAAGAaT^a^^,^ b ^,^ c	40	199
	rs1219648-323T-2A			
	Forward outer primer:	AGGTGACACTATAGAATACACAATGGCGCAGAATTA^a^	6.5	
	rs1219648-162			
	Reverse outer primer:	GTACGACTCACTATAGGGACTGGACAGGTCATTGTGGTG^a^	7	
	rs1219648-509			
rs3803662	Forward inner primer(C):	AGGTGACACTATAGAATACTCTCCTTAATGCCTCTATAGCTGTaC^a^^,^ b ^,^ c	45	259
	rs3803662-275C-2A			
	Reverse inner primer(T):	GTACGACTCACTATAGGGACCACAGTTTTATTCTTCGCTAAcA^a^^,^ b ^,^ c	80	357
	rs3803662-324A-2C			
	Forward outer primer:	AGGTGACACTATAGAATAGTCCTTGGCTGTTCTGTGA^a^	11.5	
	rs3803662-5			
	Reverse outer primer:	GTACGACTCACTATAGGGATCCCCAAGGAGACAAAGGTA^a^	7	
	rs3803662-496			
rs889312	Forward inner primer(A):	AGGTGACACTATAGAATATGCCCCTGCTGGAGAAAGtA^a^^,^ b ^,^ c	60	213
	rs889312-382A-2T			
	Reverse inner primer(C):	GTACGACTCACTATAGGGATGATTTGTAGTCTCTTAATTTGCACAaG^a^^,^ b ^,^ c	75	340
	rs889312-428G-2A			
	Forward outer primer:	AGGTGACACTATAGAATACCTGGGTCCTTAGCATTCC^a^	11	
	rs889312-126			
	Reverse outer primer:	GTACGACTCACTATAGGGAATCTGTGCCCTGAAGTGAGTAG^a^	8.5	
	rs889312-557			
rs750749	Forward inner primer(C):	AGGTGACACTATAGAATAGAGGCTGAGAACTAATAATAATTTTcC^a^^,^ b ^,^ c	50	371
	rs750749-275C-2C			
	Reverse inner primer(T):	GTACGACTCACTATAGGGATCAGATGAAAACTGCTGTGTAAGaA^a^^,^ b ^,^ c	75	224
	rs750749-325A-2A			
	Forward outer primer:	AGGTGACACTATAGAATACCCACAGAGTTGGAATGCTT^a^	11	
	rs750749-139			
	Reverse outer primer:	GTACGACTCACTATAGGGATGCATAAAGTGGGTCCTGC^a^	7.5	
	rs750749-608			
rs749292	Forward inner primer(G):	AGGTGACACTATAGAATACCTTCTTCAAACCTCGGAGTaG^a^^,^ b ^,^ c	35	282
	rs749292-429G-2A			
	Reverse inner primer(A):	GTACGACTCACTATAGGGAGATAGAAATTGTGCAGGAATCCaT^a^^,^ b ^,^ c	25	330
	rs749292-472T-2A			
	Forward outer primer:	AGGTGACACTATAGAATATCCACGGACAGAGCAGG^a^	4.5	
	rs749292-180			
	Reverse outer primer:	GTACGACTCACTATAGGGAGATTAGGGGACCTCCGTGA^a^	6	
	rs749292-673			
Tag-F		AGGTGACACTATAGAATA^d^	500	
Tag-R		GTACGACTCACTATAGGGA^d^	500	

a Underlined is the universal tag sequence at the 5′ end of the chimeric primers.

b Allele-specific nucleotides at the 3′ end are indicated in bold letters.

c Specificity is increased by the introduction of a deliberate mismatch at position -1 of the polymorphism site, indicated by bold lower case letters.

d A pair of universal primers annealing to the 5′ portion of each chimeric primer.

Genotyping of the assayed polymorphisms was performed by multiplex PCR amplification and fragment analysis. Six sets of T-ARMS-PCR primers for the amplification of twelve fragments of different sizes were pooled in a single 20 µl of reaction volume, which also contained 10 µL master mix of QIAgen Multiplex PCR kit, 50–100 ng of genomic DNA, and optimized concentrations of each primer (see Table 1). Multiplex PCR was performed using Bioer LifePro Thermal Cycler. An optimized temperature switch PCR (TSP) protocol, which uses four different annealing temperature was performed as follows: initial denaturation step of 95°C for 10 min, 3 cycles of 95°C for 30 s, 60°C for 30 s, and 72°C for 45 s, 10 cycles of 95°C for 30 s, 58°C for 30 s, and 72°C for 45 s, 20 cycles of 95°C for 30 s, 68°C for 30 s, and 72°C for 45 s, 15 cycles of 95°C for 30 s, 55°C for 30 s, and 72°C for 45 s, followed by a final extension cycle at 72°C for 10 min, and then cooled to 4°C.

The multiplex PCR products were separated by QIAxcel^®^ DNA high-resolution gel cartridge (Qiagen) on QIAxcel system (Qiagen). DNA Size Marker of 25–450 bp (Qiagen) and Alignment Marker 15 bp/500 bp (Qiagen) were used in each QIAxcel runs and the size of the products was determined using the ScreenGel software (Qiagen). Because each of the amplicons was of a different length, the alleles were detected on the basis of the patterns of peak sizes.

A total of 186 samples were also sequenced in parallel with an ABI 3130 Genetic Analyzer (Applied Biosystems, USA) according to the BigDye Termination version 3.1 protocol in Invitrogen Corporation (Shanghai, China) to confirm the multiplex T-ARMS-PCR results using the outer primers listed in table 1 for each SNP.

## Results

**Figure 1:**
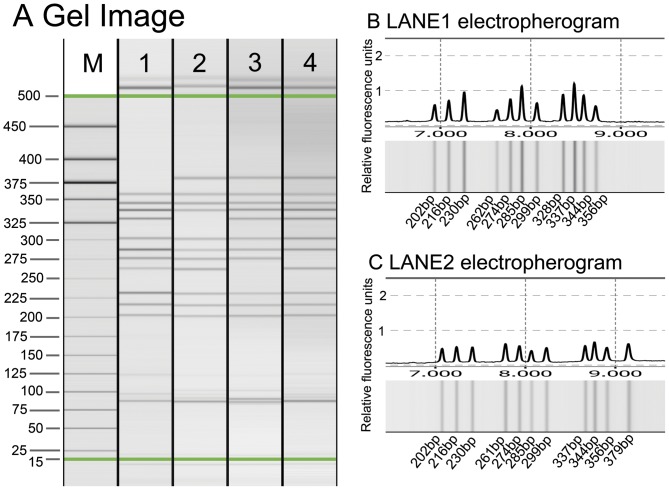
Electrophoresis result of 4 samples with most number of amplicons. A: gel image of the 4 samples. B. Gel image and electropherogram of sample in lane1. 11 bands ranging from 202 to 356 bp were observed, indicating 5 heterogenous and 1 homogenous SNPs were identified C: Gel image and electropherogram of sample in lane2. Another combination of 5 heterogenous and 1 homogenous SNPs were observed.

**Figure 2:**
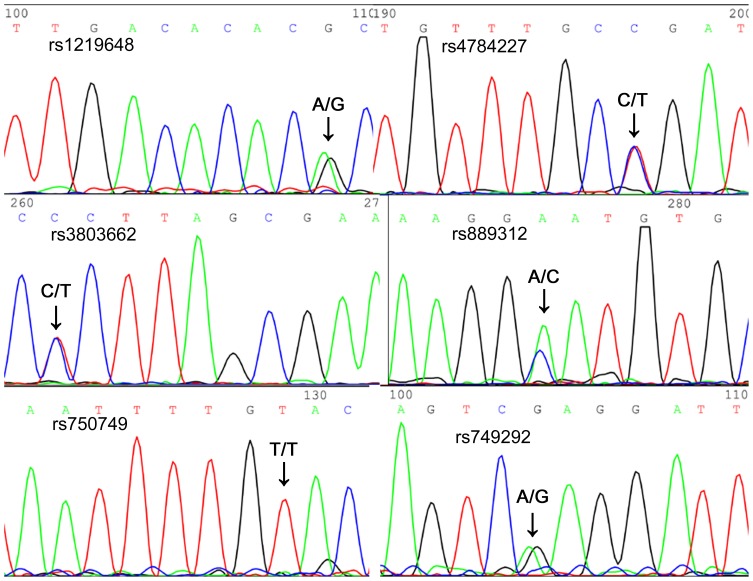
Direct sequencing result of sample #1101–291(corresponding to the sample in lane 1, Fig.1B), which showed the combination of 5 heterogenous and 1 homogenous SNPs.

A total of 186 samples were typed with a multiplex T-ARMS-PCR assay and also typed in parallel with direct sequencing to assess the accuracy and efficiency of the assay. All the PCR products were well resolved and sized by CE and ScreenGel, allowing the easy identification of different genotypes. Two of the 186 samples had eleven unique amplicons, meaning those patients have only one homogenous SNP among the six tested loci. Electropherogram and gel image of these two samples (Fig. 1) show that CE on QIAXEL can clearly separate as many as 11 fragments in one sample. The accuracy of multiple PCR analysis of one sample was confirmed by direct sequencing (Fig. 2). Fragment sizes determined by QIAXEL based CE are listed in Table 2. Read lengths were 2 to 10 bp larger than the expected ones but this did not interfere with allele determination. Heterozygotes and homozygotes were unambiguously assigned from the CE profiles. No cross-reaction was observed. The genotypes scored from the multiplex assay were in 100% accordance with direct sequencing.

**Table 2:** Analyses of read lengths in electrophoresis.

	Theoretical length	Min. read length	Max. read length	Average read length	Sd.of read length	99% CI of mean
rs1219648-A(199)	199	201	206	203.09	1.18	(200–206)
rs889312-A(213)	**213**	**215**	**220**	**216.48**	**1.17**	**(213**–**219)**
rs750749-T(224)	224	229	234	231.21	1.13	(228–234)
rs3803662-C(259)	**259**	**259**	**265**	**261.98**	**1.09**	**(259**–**265)**
rs1219648-G(269)	269	273	277	274.74	1	(272–277)
rs749292-G(282)	**282**	**282**	**289**	**285.91**	**1.12**	**(283**–**289)**
rs4784227-C(297)	297	298	305	300.16	1.02	(298–303)
rs4784227-T(322)	**322**	**327**	**330**	**328.39**	**0.8**	**(326**–**330)**
rs749292-A(330)	330	336	339	337.5	0.63	(336–339)
rs889312-C(340)	**340**	**342**	**347**	**344.73**	**0.82**	**(343**–**347)**
rs3803662-T(357)	357	352	360	356.1308	1.34	(353–360)
rs750749-C(371)	**371**	**377**	**382**	**379.3372**	**0.86**	**(377**–**382)**

The genotype distribution and allele frequencies of each SNP are listed in table 3. The allele reported to be associated with risk of cancer occurrence is highlighted. The observed frequency of genotypes in this study was overall similar to that measured by HapMap for a Han Chinese population (HCB). If the HapMap frequencies were used to predict expected genotype counts in this study, then a comparison of this population to the HapMap HCB population showed significant divergence for rs4784227 in Tox3 and rs749292 in CYP19A1, in which the risk and non-risk alleles, respectively, are present at a significantly higher proportion than previously reported for a Chinese population. It is likely there is diversity within the Han population that is not yet captured by the HapMap studies. A supplementary table (Table S1) is provided to show the exact sets of alleles for all 6 SNPs found in each individual sample for further reference.

**Table 3:** Genotypes and allele frequencies of tested samples in this study.

	genotype	N (n = 186)	Frequency (%)	Expected genotype frequency under HWE(%)	HapMap frequency HCB (%)	Allele^a^	Allele frequency (%)	HW (*p*-value)^b^
rs4784227	CC	93	50.00	51.52	66.7	C	71.77	0.31
	CT	81	43.55	40.52	24.4	**T**	28.23	
	TT	12	6.45	7.97	8.9			
rs1219648	AA	66	35.48	34.66	40	A	58.87	0.64
	AG	87	46.77	48.43	46.7	**G**	41.13	
	GG	33	17.74	16.92	13.3			
rs3803662	CC	13	6.99	8.59	6.7	C	29.30	0.29
	CT	83	44.62	41.43	44.4	**T**	70.70	
	TT	90	48.39	49.98	48.9			
rs889312	CC	41	22.04	22.64	27.3	**C**	47.58	0.74
	AC	95	51.08	49.88	45.5	A	52.42	
	AA	50	26.88	27.48	27.3			
rs750749	TT	143	76.88	77.74	80	**T**	88.17	0.26
	CT	42	22.58	20.86	17.8	C	11.83	
	CC	1	0.54	1.40	2.2			
rs749292	GG	62	33.33	30.07	8.9	G	54.84	0.07
	GA	80	43.01	49.53	55.6	**A**	45.16	
	AA	44	23.66	20.40	35.6			

a increased risk allele in bold

b *p* value was calculated using chi test

## Discussion

Methods allowing low-cost, fast and reliable SNP determination are attracting increasing interest in the age of personalized medicine. It is widely acknowledged that using information from multiple SNP genotypes provides a more accurate risk assessment than that predicted by a single risk allele[29], therefore methods capable of identifying multiple genotypes, such as MALDI-TOF mass spectrometry [30], [31] and hybridization-based [32]–[34] or enzyme-based[35] methods have been utilized. However these methods either require costly special equipment, such as mass spectrometer, or time consuming post-PCR operation.

Tetra-primer ARMS-PCR method has become one of the most commonly used methods for SNP genotyping. It requires only regular molecular biology equipment and eliminates the need of hybridization or additional enzymatic reactions. Although triplex and quadruplex PCR methods have been reported, there is limited use of multiplex T-ARMS-PCR in genotyping because of two key limitations. Firstly, the probability of finding primers with matched melting temperatures drastically drops when trying to combine the detection of several SNPs in a single reaction. Secondly, the resulting pool of amplicons requires sufficient length intervals between neighboring bands in electrophoresis to facilitate separation. By combining T-ARMS-PCR with a chimeric primer-based temperature switch PCR strategy we largely sidestep these limitations. Our method demonstrates for the first time the ability of tetra-primer ARMS-PCR to easily detect six SNPs in a single reaction.

The reliability of the method was illustrated by typing 186 clinical blood samples in parallel with direct sequencing, and a 100% consistency between the two methods was obtained. This proof-of-concept study thus establishes a rapid, reproducible, and cost effective method for the detection of multiplex SNPs, since CE by QIAXCEL is capable of resolving amplicons with as little as 5 bp size difference, the smallest size difference in this test was 10 bp. As the read lengths in this assay has a standard deviation of 0.8 to 1.3 (Table 2), determination of allele is thus not interfered.

The use of chimeric primers and biphasic temperature switch in the annealing process decreases the difference in amplification efficiency among amplicons. During the first few PCR cycles, amplification is carried out by allele-specific chimeric primers. In later stages of PCR, amplification is predominantly carried out by universal primers, so that all targets in this multiplex PCR system are amplified in an unbiased manner by a single pair of universal primers. This reduces the occurrence of biased and partial amplification, minimizes non-specific reactions, and reduces the need for optimization of each individual PCR assay.

To assess the errors resulting from the utilization of multi-band electrophoregrams to distinguish amplicons, the read length of each band was compared with the theoretical lengths calculated from primer-alignment (Table 2). No overlapping in read length range between any two of the amplicons was observed, additionally, there is no overlap of the 99% confidence intervals of the observed average read length. It is notable that band intensities can be subject to several factors including genomic DNA quality and PCR reagents quality; we have found that 50–100 ng/µL DNA is optimal to provide sufficiently clear and bright bands with minimal background (data not shown). In addition, unlike most other reported T-ARMS-PCR methods, a deliberated mismatch at position -1 from the 3′ terminus was incorporated into both inner primers and was sufficiently specific for the differential detection of two alleles for each SNP. Due to the limitation of size discrimination between PCR products, PCR primer design may be restricted to some extent, making multiple T-ARMS-PCR difficult to type those SNPs that are located closer than 20 bp to each other.

Two distinct advantages of the multiple ARMS-PCR method are the short assay time and the low costs, even for assaying large numbers of specimens. The proposed method, only involving conventional PCR with a CE, can be performed within 3.5 hours with minimal hands-on effort. After extraction of genomic DNA, the subsequent steps can be completed in a single reaction tube, allowing for the ready analysis of multiple samples in a single run for high-throughput screening. This assay consumes only standard PCR reagents and electrophoresis cartridges; the costs in this study were only 2 US$ for the simultaneous detection of six SNPs per sample.

To our knowledge, the proposed method is the first to detect six SNPs in a single reaction using tetra-primer ARMS-PCR. The novel multiplex tetra-primer ARMS-PCR method developed in this study has significant potential to be widely applicable in both commercial and clinical settings for the screening of multiple SNPs.

## Supporting Information

Table S1 **Alleles of 6 SNPs of each individual sample in this study.**
(DOCX)
