# Supplementary material for: A Novel Multiplex Tetra-Primer ARMS-PCR for the Simultaneous Genotyping of Six Single Nucleotide Polymorphisms Associated with Female Cancers
Source: PLoS One. 2013 Apr 17;8(4):e62126. doi: 10.1371/journal.pone.0062126 (PMC3629144; doi:10.1371/journal.pone.0062126)
Supplement: Table S1 — Alleles of 6 SNPs of each individual sample in this study. (DOCX) [file pone.0062126.s001.docx]

Supplement table 1. Genotyping results of all 6 SNPs in each individual (n=185) from direct sequencing*

| Sample ID | rs1219648 | rs889312 | rs3803662 | rs750749 | rs749292 | rs4784227 |
| --- | --- | --- | --- | --- | --- | --- |
| w76 | AA | CC | CT | TT | GA | CT |
| w75 | GG | AC | CT | TT | GA | CC |
| w74 | AG | AC | TT | TT | GA | CC |
| w73 | GG | CC | CT | TT | GA | CT |
| w72 | AG | CC | CT | TT | GA | CT |
| w71 | AG | AA | CT | TT | GA | CT |
| w70 | AA | AA | TT | TT | GG | CT |
| w62 | AG | AC | CC | TT | GG | CC |
| w61 | AG | CC | TT | TT | GA | CC |
| w59 | AG | AA | CT | TT | AA | CT |
| w57 | AG | AA | TT | TT | GA | CC |
| w48 | GG | AC | TT | CT | GG | CC |
| w47 | AG | AC | TT | TT | GA | CC |
| w46 | AG | CC | CT | TT | GG | CT |
| w45 | AA | AC | CT | TT | GA | CC |
| w44 | AA | AC | CT | TT | GG | CC |
| w43 | AG | AA | CT | CT | GA | CC |
| w42 | AG | AC | CT | TT | AA | CC |
| w41 | AA | AA | TT | CT | AA | CT |
| w40 | AA | AC | CT | CT | GA | CT |
| w39 | AG | AC | TT | TT | GG | CC |
| w38 | GG | AA | CT | TT | AA | CT |
| w37 | AG | CC | TT | CT | AA | CT |
| w36 | AA | AA | CT | CT | GA | CC |
| w35 | AG | AA | TT | TT | GA | CC |
| w32 | AA | CC | CT | TT | AA | CC |
| w28 | AG | AC | TT | TT | GA | CT |
| w27 | AG | AC | CT | TT | GG | CT |
| w25 | AG | AA | CT | TT | AA | CT |
| w21 | AG | AA | TT | TT | GA | CC |
| w20 | AG | AA | CT | TT | GA | CC |
| w19 | AG | AC | TT | TT | GG | CT |
| w17 | AA | CC | CT | TT | GG | CC |
| w16 | GG | AC | CT | TT | AA | CT |
| w15 | AA | AA | CT | CT | GG | CT |
| w14 | AG | AC | CC | TT | GA | CC |
| w13 | AA | AC | CT | TT | GA | CC |
| w1 | AG | CC | TT | CT | GG | CC |
| cs14 | AA | CC | CC | CT | GA | CC |
| cs13 | AA | CC | CT | TT | GG | CC |
| 1102-62 | GG | AC | CT | TT | GA | CT |
| 1102-37 | AA | AC | CT | TT | GG | CC |
| 1102-178 | AG | AC | TT | CT | GG | TT |
| 1102-040 | AA | AC | TT | CT | AA | CT |
| 1101-295 | AA | AC | CT | TT | GA | CT |
| 1101-291 | AG | AC | CT | TT | GA | CT |
| 1101-290 | AA | AC | CC | TT | GG | CC |
| 1101-279 | AA | CC | CT | CT | GA | CT |
| 1101-277 | AA | CC | TT | CT | AA | CT |
| 1101-274 | AA | AC | TT | TT | GA | CT |
| 1101-272 | AG | CC | CT | TT | GG | CC |
| 1101-269 | GG | AC | CT | TT | GA | CT |
| 1101-267 | AA | AA | TT | CT | GG | CC |
| 1101-266 | AA | AC | TT | CT | AA | CC |
| 1101-264 | AA | AC | CT | TT | GA | CC |
| 1101-259 | AG | AA | TT | TT | AA | CT |
| 1101-244 | AA | CC | TT | CT | AA | CT |
| 1101-243 | AA | AC | CT | TT | GG | CC |
| 1101-238 | AG | AC | CT | TT | GG | CT |
| 1101-232 | AG | AA | TT | CT | GG | CC |
| 1101-227 | AA | AA | CT | CT | GG | CT |
| 1101-226 | GG | AC | TT | CT | GA | CC |
| 1101-225 | AG | AC | TT | TT | GA | CT |
| 1101-223 | AG | AC | CC | TT | AA | CT |
| 1101-222 | AG | AA | TT | CT | GG | CT |
| 1101-218 | GG | AC | TT | TT | GG | CT |
| 1101-217 | AA | AC | TT | CT | GA | TT |
| 1101-216 | AG | CC | TT | TT | GA | TT |
| 1101-215 | GG | CC | CT | TT | GA | CC |
| 1101-214 | GG | CC | TT | TT | GG | CT |
| 1101-213 | GG | AC | TT | CT | AA | CC |
| 1101-212 | AG | AA | TT | TT | GA | CC |
| 1101-211 | AA | AC | TT | TT | GG | CT |
| 1101-197 | AA | AC | CT | CT | AA | CC |
| 1101-193 | AA | AC | CT | CT | AA | CC |
| 1101-180 | AA | CC | TT | CT | GG | CC |
| 1101-172 | AG | CC | TT | TT | AA | CT |
| 1101-169 | AG | CC | TT | TT | GG | CT |
| 1101-167 | AA | AA | CT | TT | GA | CT |
| 1101-162 | AG | AA | CT | TT | GG | CC |
| 1101-161 | AA | CC | CT | TT | GG | CC |
| 1101-153 | GG | AA | TT | TT | GG | CT |
| 1101-152 | AG | AC | TT | TT | GA | CT |
| 1101-151 | AA | AC | TT | TT | AA | CT |
| 1101-135 | AG | AC | TT | CT | GG | CC |
| 1101-130 | AG | AC | CT | TT | AA | CT |
| 1101-127 | AA | CC | CT | TT | AA | CC |
| 1101-124 | GG | AC | CC | TT | GA | CC |
| 1101-116 | GG | AA | TT | TT | GA | TT |
| 1101-114 | AG | AA | CT | CT | GG | CC |
| 1101-107 | AA | CC | TT | TT | GG | TT |
| 1101-106 | GG | CC | CT | TT | GA | CC |
| 1101-092 | AA | AA | CT | TT | GG | CC |
| 1101-084 | AG | AC | TT | TT | GG | CT |
| 1101-082 | AA | AC | TT | TT | GA | CT |
| 1101-081 | AG | AC | CT | TT | GA | CC |
| 1101-073 | AA | AC | CC | TT | AA | CC |
| 1101-071 | AG | AA | TT | TT | GG | TT |
| 1101-069 | AG | AA | CT | CT | AA | CC |
| 1101-068 | AG | AC | TT | CT | GA | CT |
| 1101-067 | AG | AA | TT | TT | GG | CT |
| 1101-066 | AA | AC | CT | CT | GG | CT |
| 1101-062 | AA | AA | TT | TT | GA | CC |
| 1101-059 | AG | AC | CT | TT | GG | CT |
| 1101-055 | AG | AC | TT | TT | AA | CT |
| 1101-053 | AA | AA | TT | TT | AA | CT |
| 1101-052 | AG | AC | TT | TT | GG | CT |
| 1101-051 | GG | CC | TT | TT | AA | CT |
| 1101-049 | AA | AC | TT | TT | AA | CT |
| 1101-046 | AA | AC | CT | TT | AA | CT |
| 1101-044 | AA | AA | CT | TT | GA | CC |
| 1101-042 | AG | CC | TT | TT | GG | CT |
| 1101-041 | AG | AC | CT | CT | GG | CC |
| 1101-038 | AA | AC | TT | CT | GG | CT |
| 1101-037 | AG | AC | TT | TT | GA | CC |
| 1101-036 | AG | AA | TT | TT | GG | CT |
| 1101-032 | AA | AA | TT | TT | GG | CC |
| 1101-030 | AG | CC | TT | TT | GG | CT |
| 1101-028 | AA | AC | TT | TT | AA | CT |
| 1101-023 | AG | AC | TT | TT | GA | CT |
| 1101-022 | AG | AC | CT | TT | GA | CC |
| 1101-021 | AG | AC | CT | TT | GG | CC |
| 1101-020 | GG | CC | CT | TT | GA | CC |
| 1101-018 | AG | AC | CT | TT | GG | CC |
| 1101-017 | GG | AA | TT | CC | GA | CC |
| 9230 | AG | AC | CT | TT | GA | CC |
| 9179 | AG | AC | TT | CT | AA | CT |
| 9055 | GG | AC | CT | TT | AA | CC |
| 9029 | AA | CC | CC | CT | GA | CC |
| 9008 | AA | CC | TT | TT | GG | CT |
| 9007 | AA | AC | CC | TT | GA | CC |
| 9006 | AG | AA | TT | CT | AA | CC |
| 9005 | GG | AA | TT | TT | GG | TT |
| 8964 | AA | CC | TT | TT | AA | CC |
| 8963 | AG | AA | TT | TT | GA | CC |
| 8962 | AG | AC | TT | TT | GG | CT |
| 8961 | AG | AA | CT | TT | GA | CC |
| 8940 | AG | AC | CT | CT | GA | CC |
| 8939 | GG | AA | CT | TT | GG | CC |
| 8918 | AA | AC | TT | CT | GA | CT |
| 8917 | GG | AC | CC | TT | AA | CC |
| 8891 | GG | AC | TT | TT | GA | CT |
| 8891 | GG | AC | TT | TT | GA | CT |
| 8889 | AG | AA | CT | TT | GG | CC |
| 8824 | GG | AC | CT | TT | AA | CC |
| 8823 | AG | AC | CC | CT | GA | CC |
| 7903 | GG | AA | TT | TT | GA | TT |
| 7901 | AG | AC | CT | TT | GG | CT |
| 7599 | AA | CC | CT | TT | GA | CC |
| 7598 | AG | CC | TT | TT | AA | TT |
| 7597 | AG | AC | CC | TT | AA | CC |
| 7578 | AG | AA | TT | TT | GA | CC |
| 7577 | AG | AC | TT | TT | GG | TT |
| 7576 | AG | AA | TT | TT | GA | CC |
| 7466 | AA | CC | CT | TT | GG | CC |
| 7465 | AA | AC | CT | TT | GG | CT |
| 7444 | GG | AC | CT | TT | GA | CT |
| 7291 | AG | AA | TT | TT | AA | TT |
| 7285 | AG | CC | TT | TT | GG | CT |
| 7013 | AG | CC | TT | CT | GG | CT |
| 7012 | AA | AA | CT | TT | GA | CC |
| 7011 | AG | AA | TT | TT | GA | CT |
| 7010 | AG | AA | CT | TT | AA | CC |
| 7009 | AG | AC | CT | TT | GA | CC |
| 7008 | GG | AC | TT | TT | GA | CT |
| 7007 | GG | AC | TT | TT | GA | CT |
| 7006 | AG | AC | CT | TT | GA | CC |
| 7005 | AA | AC | TT | TT | AA | CT |
| 7004 | AA | AC | CT | TT | GA | CC |
| 7003 | AG | CC | TT | TT | GG | TT |
| 7002 | AA | AC | CT | TT | GA | CC |
| 7001 | AA | CC | TT | TT | GA | CT |
| 6217 | GG | AC | CT | TT | GA | CC |
| 6216 | GG | AC | CC | TT | AA | CC |
| 6199 | AA | AC | CT | CT | GA | CT |
| 6198 | AA | AC | CT | CT | GA | CT |
| 5604 | AA | AC | CT | TT | AA | CC |
| 5602 | AG | AA | CT | TT | GA | CC |
| 5586 | AG | CC | CT | TT | AA | CC |
| 5582 | AG | AC | CT | TT | GA | CC |
| 5575 | AG | AC | TT | TT | AA | CC |
| 5572 | AG | AC | TT | TT | GG | CT |
| 5453 | GG | AA | CT | TT | GA | CC |
| 5450 | AA | AC | TT | TT | GA | CT |
| 5448 | AG | CC | TT | TT | GA | CT |
| 5446 | AA | AA | CT | CT | GG | CC |

*: All results obtained from T-ARMS-PCR coincide completely with direct sequencing
